# Helper T Lymphocyte Response in the Peripheral Blood of Patients with Intraepithelial Neoplasia Submitted to Immunotherapy with Pegylated Interferon-α

**DOI:** 10.3390/ijms16035497

**Published:** 2015-03-10

**Authors:** Márcia Antoniazi Michelin, Letícia Montes, Rosekeila Simões Nomelini, Marco Aurélio Trovó, Eddie Fernando Candido Murta

**Affiliations:** 1 Oncology Research Institute (IPON), Federal University of the TriânguloMineiro (UFTM), Uberaba, Minas Gerais 38022-200, Brazil; E-Mails: michelinimuno@icbn.uftm.edu.br (M.A.M.); letsmontes@yahoo.com.br (L.M.); 2 Discipline of Immunology, UFTM, Uberaba, Minas Gerais 38022-200, Brazil; 3 Discipline of Gynecology and Obstetrics, UFTM, Uberaba, Minas Gerais 38022-200, Brazil; E-Mails: rosekeila@terra.com.br (R.S.N.); marcotrovo@yahoo.com.br (M.A.T.)

**Keywords:** immunotherapy, cervical cancer, IFN-α and T lymphocytes

## Abstract

Immunotherapy in cancer patients is a very promising treatment and the development of new protocols and the study of the mechanisms of regression is imperative. The objective of this study was to evaluate the production of cytokines in helper T (CD4^+^) lymphocytes during immunotherapy with pegylated IFN-α in patients with cervical intraepithelial neoplasia (CIN). We conducted a prospective study with 17 patients with CIN II-III using immunotherapy with pegylated IFN-α subcutaneouly weekly, and using flow cytometry we evaluated the peripheric CD4^+^ T lymphocytes. The results show that in the regression group the patients presented a significant increase in the amount of IFN-γ during the entire immunotherapy, compared with the group without a response. The amount of CD4^+^ T lymphocytes positive for IL-2, IL-4, IL-10 and TGF-β is significantly lower in patients with good clinical response. The results also demonstrate that patients with regression have a higher amount of intracellular TNF-α in CD4^+^ T lymphocytes before the start of treatment. Analyzing these data sets, it can be concluded that immunotherapy is a viable clinical treatment for patients with high-grade CIN and that the regression is dependent on the change in the immune response to a Th1 pattern.

## 1. Introduction

Cervical intraepithelial neoplasia (CIN) is a precursor lesion to cervical cancer. An estimated 500,000 women worldwide are diagnosed with CIN annually. For the last 30 years, scientists have studied the association between cervical cancer and infection with human papillomavirus (HPV) [1]. Viral DNA from HPV has been found in about 90% of cervical cancer biopsies [2]. Certain HPV viral proteins interfere with the transmission of proteins and genes related to the immune response, such as E6 and E7, which block cytokine synthesis [3].

Presently, the conventional treatment of high-grade lesions (CIN II/III) is the ablative excisional methods (laser) or conization [4]. However, several studies have indicated that women who have had these procedures have some obstetric complications, the most common being preterm delivery, low birth weight and premature rupture of membranes [5,6].

Clinical and experimental data have shown that local and systemic cytokines can induce tumor regression, mainly those that could induce a cellular immune response. For patients with CIN II/III or other types of neoplasia, conservative treatment with IFN-α can preserve the woman’s reproductive ability by avoiding the need for more invasive procedures, such as conization. Interferons (IFNs) are glycoproteins that were initially described with respect to their strong antiviral effects [7]. A study by Ramos *et al.* [8] demonstrated that IFN-α–treated patients with CIN III and with tumor regression expressed more type 1 T helper (Th1)-profile cytokines (IFN-γ, Tumor necrosis factor (TNF)-α, interleukin (IL)-2), with a significant reduction in the high-risk HPV viral load in the lesions. Patients whose therapy failed were smokers and had a higher expression of Th2-type (IL-4) or regulatory T cytokines (transforming growth factor (TGF)-β2 and TGF-β3).

Immune response studies are important for understanding the systemic responsiveness to endogenous IFN-α in patients with CIN III, as well as for developing new therapeutic protocols. Therefore, the objective of this study was to evaluate the production of cytokines (IL-2, TNF-α, IFN-γ, TGF-β, IL-10, IL-4, IL-17, IL-12), IL-2 receptor (CD25) and transcription factor (FOXP3) in helper T (CD4^+^) lymphocytes during the treatment of patients with CIN II/III with pegylated IFN-α.

## 2. Results

**Figure 1:**
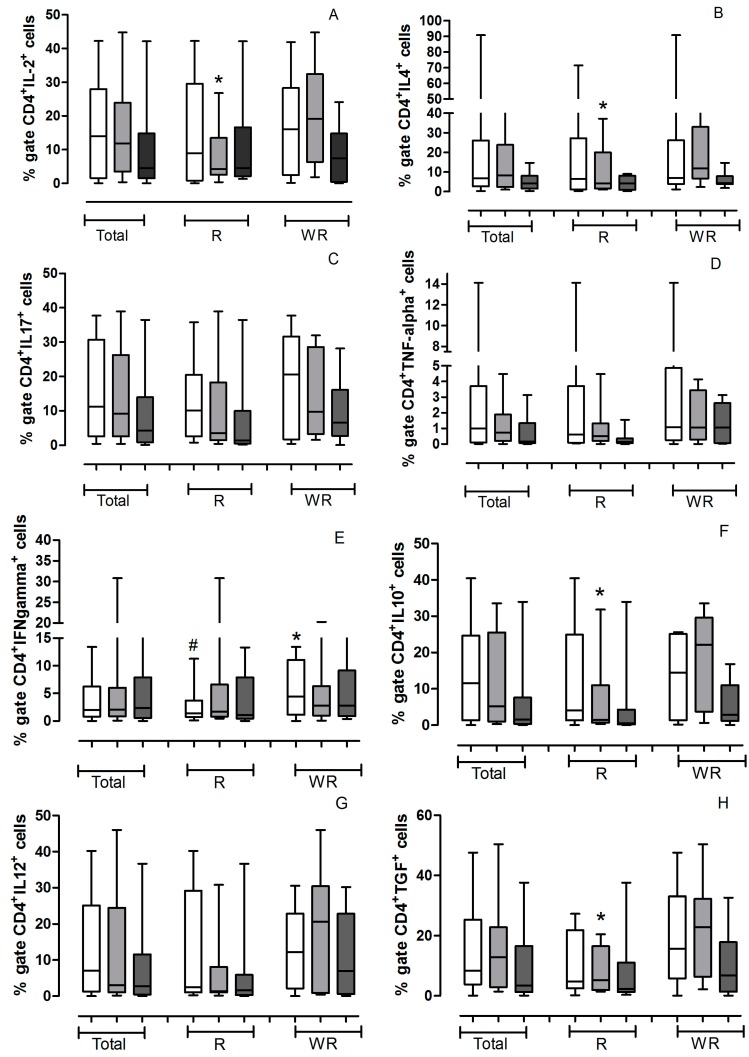

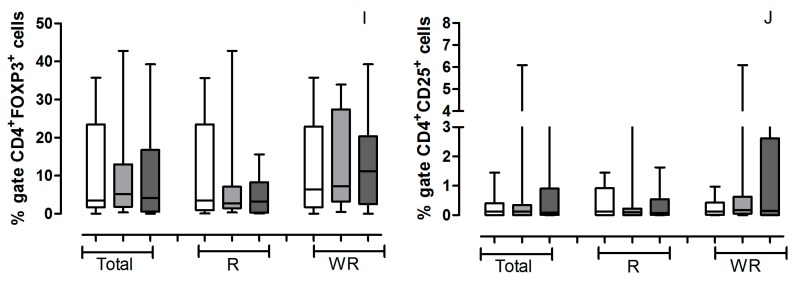
Values (median) of % gate of helper T lymhocytes positive for cytokines obtained from all the patients studied. The gate was determined for localization of cells corresponding to lymphocytes by relative size (Forward Scatter, FSC) and granularity/complexity (Side Scatter, SSC) in each experiment and for each patient, as shown in Figure 3. The helper lymphocytes marked with CD4^+^ and cytokines, receptor or transcription factor antibodies were analyzed in the pretreatment (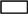) and in the 3th (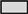) and 6th (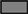) applications. The analyses were conducted with all patients studied (Total) and separately according to treatment outcome (Regression—R or Without Regression—WR). All values are expressed in Median. (**A**) CD4^+^IL-2^+^ * R3a *vs.* WR3a *p* = 0.0592; (**B**) CD4^+^IL-4^+^ * R3a *vs.* WR3a *p* = 0.0927; (**C**) CD4^+^IL-17^+^; (**D**) CD4^+^TNF-α^+^; (**E**) CD4^+^IFN-γ^+^ # RPre *vs.* WR pre *p* = 0.0274; * WRPre *vs.* WR6a *p* = 0.0366; (**F**) CD4^+^IL-10^+^ * R3a *vs.* WR3a *p* = 0.0745. (**G**) CD4^+^IL-12^+^; (**H**) CD4^+^TGF-β^+^ * R3a *vs.* WR3a *p* = 0.0464; (**I**) CD4^+^FOXP3^+^; (**J**) CD4^+^CD25^+^.

**Figure 2:**
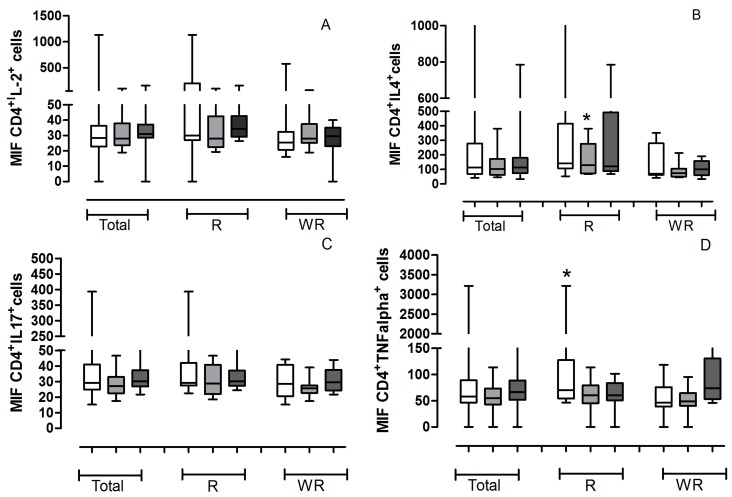

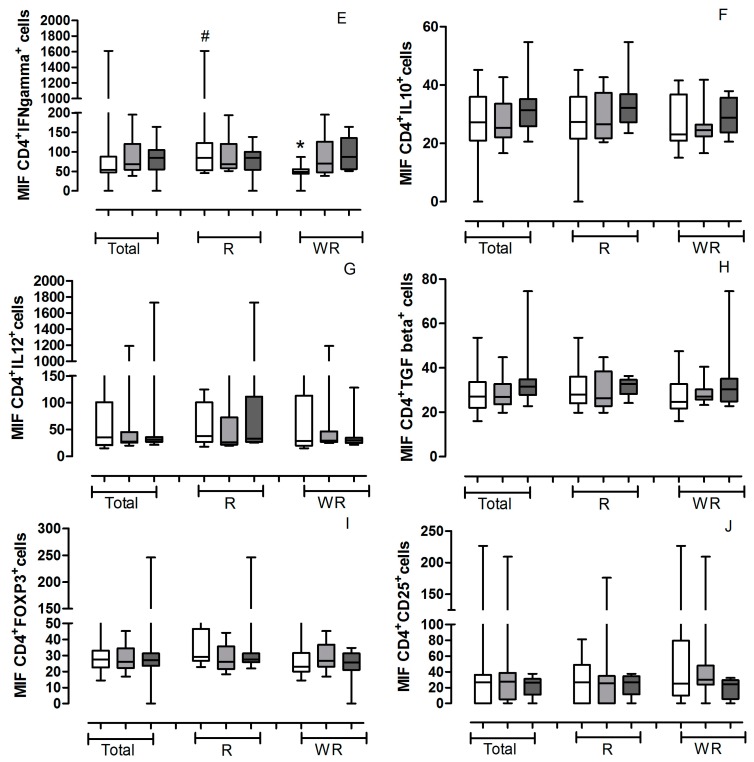
Values of median of intensity of fluorescence (MFI) of helper T lymphocytes positive for cytokines obtained from all the patients studied. The gate was determined for localization of cells corresponding to lymphocytes by for relative size (Forward Scatter, FSC) and granularity/complexity (Side Scatter SSC) in each experiment and for each patient. The helper lymphocytes marked with CD4^+^ and cytokines, receptor or transcription factor antibodies were analyzed in the pretreatment (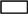) and in the 3th (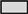) and 6th (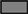) applications. The analyses were conducted with all patients studied (Total) and separately according to treatment outcome (Regression or Without Regression). All values are expressed in Median. (**A**) CD4^+^IL-2^+^; (**B**) CD4^+^IL-4^+^ * R3a *vs*. WR3a *p* = 0.0745; (**C**) CD4^+^IL-17^+^; (**D**) CD4^+^TNF-α^+^ * Rpre *vs*. WRpre *p* = 0.0464; (**E**) CD4^+^IFN-γ^+^ # Rpre *vs*. WRpre *p* = 0.0927; * WRpre *vs*. WR6a *p* = 0.0042; (**F**) CD4^+^IL-10^+^; (**G**) CD4^+^IL-12^+^; (**H**) CD4^+^TGF-β^+^; (**I**) CD4^+^FOXP3^+^; (**J**) CD4^+^CD25^+^.

We evaluated the expressions cytokines, IL-2, IL-4, IL-17, TNF-α, IFN-γ, IL-10, IL-12, TGF-β, IL-2 receptor (CD25) and FOXP3 in helper T lymphocytes (CD4^+^). The data are presented as median of % Gate (number of cells positive for each marker) (Figure 1) or intensity of Fluorescence (MFI, quantity of the protein, independent of the quantity of positive cells) (Figure 2) obtained from all the patients studied.

The results demonstrate that patients with regression by immunotherapy have a significant reduction in the number of CD4^+^lymphocytes positive for IFN-γ (*p* = 0.0274) (Figure 1E), however, those lymphocytes have a MFI IFN-γ^+^ a much higher (*p* = 0.0927) (Figure 2E) than patients without regression, demonstrating a greater capacity for synthesis of this cytokine. The Kruskal-Wallis test showed that in the group of regression the patients present a significant increase in the amount of IFN-γ during the entire immunotherapy (Figure 1 and Figure 2E).

Also regarding the production of IFN-γ by helper lymphocytes, we found that patients who do not have clinical response show a significant decrease during immunotherapy (*p* = 0.0274) (Figure 1E), however, there is an increased amount of intracellular cytokine (*p* = 0.004) (Figure 2E).

The amount of CD4^+^ T lymphocytes positive for IL-2, IL-4, IL-10 and TGF-β is significantly lower in patients with good clinical response, when compared with patients without regression in the third application of IFN-α (Figure 1A,B,F,H). However, the double positive CD4^+^ lymphocytes IL-4^+^ have an increased amount of this cytokine compared with patients with no regression (Figure 2B).

They also demonstrate that patients with regression have a higher amount of intracellular TNF-α in CD4^+^ T lymphocytes before the start of treatment than those patients without response (Figure 2D).

## 3. Discussion

Type I IFN cytokines play important biological roles within the immune system. Their main function is to induce an antiviral state in infected and adjacent cells. They also act on innate immunity by activating natural killer cells, proinflammatory pathways, and cytokine production. They activate adaptive immunity by promoting the development of an antigen-specific response through the activation of T and B lymphocytes.

The use of interferon in the treatment of CIN took place during the early 1980s and, perhaps due to differences regarding the possibility to obtain good clinical response, studies addressing this treatment were mostly restricted to that decade. However, after a few years it was found that certain cell lines were extremely sensitive to this mediator. IFN-α induces apoptosis of certain hematopoietic malignant cell lines, including melanoma cells, ovarian carcinoma cells and multiple myeloma cells [9,10,11].

The need for developing new clinical treatments for CIN has been growing in recent decades since, increasingly these cancers are present in younger patients [12], moreover pregnancy has become an increasingly late event in the life woman. Conservative therapy with pegylated interferon subcutaneously may be among the therapeutic options for patients of childbearing age since it does not alter the anatomy of the cervix, which is the factor most responsible for generating complications during pregnancy.

Since the early 1980s, several studies have used interferon in the treatment of gynecological cancer with different responses [13]. In the case of interferon-α, some studies show remission of cervical intraepithelial neoplasia, ranging from 30% to 80% of cases [14,15,16]. In the case of invasive cancer, there are reports of healing of invasive carcinoma of the vagina using intralesional interferon α-2b [17]. For some years, our group has been studying clinical remission of tumors using immunotherapy with IFN-α and its actions on the immune system. However, these results are the first to demonstrate clinical and immunological effectiveness of pegylated IFN-α in patients with CIN. We found that there are several advantages of using this pegylated cytokine, such as very minor side effects, no need for exposure of the cervix and especially reducing the number of weekly doses; and interestingly these recent data demonstrated that the administration route of IFN-α could be extremely important in the immune response; in previous publications using this immunotherapy, but administrating the cytokine by intralesional route, we demonstrated that patients whose therapy failed were smokers and had a higher expression of Th2-type (IL-4) or regulatory T cytokines (transforming growth factor (TGF)-β2 and TGF-β3) [8], however when, the IFN-α was administrated subcutaneously, we did not find a significant difference between the smoking and non-smoking groups.

Our results showed that immunotherapy with pegylated IFN-α changes the systemic pattern of cytokines produced by helper T lymphocytes. This data is in accordance with the suggestion of Scott *et al.* [18], which postulates that the viral persistence of HPV infection would occur by failure to express Th1 cytokines.

We observed that patients with clinical response to immunotherapy have a progressive increase in the synthesis of IFN-γ by helper T lymphocytes in peripheral blood. This is a major cytokine responsible for inducing a profile of cellular immune response by increasing the activity of macrophages, and inducing amplification mechanisms to drive the Th0 to Th1 lymphocytes. Several authors have demonstrated that the progression of lesion in patients with CIN is related to a reduction of systemic type TH1 interleukins (IFN-γ, TNF-α and IL-2) [19,20]. As IFN-γ, our data demonstrate that there is a significant increase of intracellular TNF-α in helper T lymphocytes of patients with a good clinical response to immunotherapy, compared with the group of patients without regression, which reinforces the hypothesis that regression of the tumor depends on the cellular immune response induction.

On the other hand, our data demonstrate that the patients who did not present a regression have higher levels of IL-4, IL-10 and TGF-β in the third application of pegylated IFN-α. Clerici *et al.* [21] and Bor-Ching *et al.* [22] hypothesize that expression of IL-4, IL-10, and TGF-β1 correlate with disease severity. Therefore, the expression of this cytokine profile would be predicted to favor a state of local immunosuppression, which is associated with deregulation of several molecules of the immune response [23].

It is also known that TGF-β is linked to the T regulatory (Treg) pathway and their production in tumors could be responsible by the induction of immunosupression. We know that IL-10 and TGF-β inhibit the maturation of dendritic cells and may indirectly inhibit cytotoxic T lymphocyte responses [24]. The Treg cells are characterized by expression of FOXP3. In our study we found an increase of CD4^+^FOXP3^+^ lymphocytes in patients without response to immunotherapy with pegylated IFN-α, however the difference in the group with response was not significant. Another interesting fact was that increased levels of IL-2 and TGF-b (Figure 1) were found in patients unresponsive to treatment. Perhaps this increase in IL-2 reflects the action of Treg cells in these patients, since this cytokine acts in an autocrine activation of all subtypes of T lymphocytes. Another fact that points us to this possibility is that in a recent work Becker *et al*. [25] demonstrated that inhibition of Treg cells by the action of IFN-α does not necessarily result in decreased of FOXP3, but inhibits the production of cyclic adenosine monophosphate (cAMP), which is directly related to the intracellular increase of the capacity of Treg cells to exert its function.

Thus, our results allow us to conclude that immunotherapy with pegylated IFN-α alters the immune profile of peripheral CD4^+^ T lymphocytes capable of inducing a local antitumor response. We can also conclude that this regression depends on a prior immune profile, which possibly depends on genetic factors and should be further investigated.

## 4. Materials and Methods

### 4.1. Patients

A prospective study was performed at the Maria da Glória outpatient clinic of the Hospital School of the Federal University of the Triângulo Mineiro, in the departments of Gynecology and Immunology from 2013 through 2014. The group studied consisted of 17 patients between 18 to 82 years of age, with diagnoses of CIN II-III, who had not received any prior treatment. Patients provided information about their ages, habits, lifestyles (e.g., smoking, use of drugs, number of partners), contraceptive methods used, history of sexually transmitted diseases, and use of hormone replacement therapy.

All procedures performed followed the criteria developed by the Ethics Committee (CEP/UFTM Nos. 759 and 1525). The inclusion criteria were: absence of bleeding during the examination; no use of oral antibiotics, vaginal fungicides or creams over the previous 30 days; no sexual activity for two days preceding sample collection; previous history of treatment for HPV; and no colposcopic change <1 cm. The exclusion criteria were: immunosuppresive diseases, serious cardiopathies, changes in liver or kidney function, pregnancy, a reported intolerance to IFN, or an absence of a visible lesion at colposcopy (Table 1).

**Table 1:** Clinical charateristics, histological diagnosis by biopsy, and conduct in each case, after immunotherapy with pegylated IFN-α.

Patient	Age	Smoker	Initial Diagnosis	Final Diagnosis	Treatment Outcome
1	36	No	CIN III	Normal Epithelium	Regression
2	67	No	NIVA III	Normal Epithelium	Regression
3	82	No	CIN III	CIN III	WithoutResponse
4	35	Yes	CIN III	CIN III	WithoutResponse
5	54	No	CIN III	Normal Epithelium	Regression
6	28	No	CIN II	Normal Epithelium	Regression
7	32	No	CIN III	CIN III	Without Regression
8	35	No	CIN III	CIN III	Without Regression
9	18	No	CIN II	CIN II	Without Regression
10	34	No	CIN II	CIN II	Without Regression
11	38	No	CIN III	CIN II	Regression
12	37	No	CIN III	CIN III	Without Regression
13	34	No	CIN III	CIN II	Regression
14	47	No	CIN III	Normal Epithelium	Regression
15	26	No	CIN III	Normal Epithelium	Regression
16	24	Yes	CIN II	CIN II	Without Regression
17	28	Yes	CIN II	Normal Epithelium	Regression

### 4.2. Application of Pegylated IFN-α

Human recombinant pegylated IFN-α 2b (Pegintron^®^; 80 mgc) was used for the therapy. The pegylated IFN-α 2b was applied via the subcutaneous abdominal vein at a dose of 80 mgc (flask-ampoule with lyophilic powder diluted in 0.7 mL of diluent before each application). The applications were performed weekly until a total of six applications were reached. Peripheral blood was collected from the vein of the right forearm of each patient prior the first injection (Pretreatment) and on 3th and 6th application.

### 4.3. Evaluation of Clinical Response

The groups evaluated in the study were based on colposcopic examination, biopsy and histology. Thus, if colposcopy showed disappearance or regression of the lesion, as confirmed by histological examination from biopsy, with regression to CIN I or no CIN, the treatment was considered as satisfactory or successful, characterizing the responsive group. The patients were submitted to follow-up with cytology and colposcopy every 6 months (Table 1).

If no regression of the lesion was observed at colposcopic examination, confirming the persistence of CIN II or III in biopsies, failure of the treatment was considered, characterizing the without response group. All patients with CIN II and III were immediately submitted to cold knife conization (Table 1).

### 4.4. Flow Cytometry

Over the course of the treatment, peripheral blood samples were collected prior (pretreatment), to the 3th and 6th application. Cells were evaluated by flow cytometry (BD FACS Calibur^®^, BD Biosciences, San Jose, CA, USA), using protocols suggested by the manufacturer (BD Biosciences^®^). Leukocytes were isolated from peripheral blood samples via centrifugation at 4 °C with a standard cell lysing protocol (FACS™Lysing Solution, BD Biosciences). Cells were resuspended in PBS. The following antibodies for extracellular proteins were added: Fluorescein isothiocyanate (FITC) or Phycoerythrin (PE)-labeled anti-CD4 (for Th cells) or CD25APC. Cells were incubated at 4 °C for 30 min, rinsed twice by centrifugation with PBS, fixed, and permeabilized with BD Cytofix/Cytoperm™ and Perm/Wash (BD Biosciences), according to the manufacturer’s protocols. For intracellular protein staining, the cells were incubated with the following FITC or PE-labeled antibodies (according to the fluorochrome extracellular antibodies) for 30 min at 4 °C: IL-2, IL-12, IL-17, IL-10, IL-4, TNF-α, TGF-β, IFN-γ or FOXP3. In all experiments and for all patients we used as negative control isotope antibodies intracellular and extracellular conjugated with fluorochromes according to the reference antibody.

**Figure 3:**
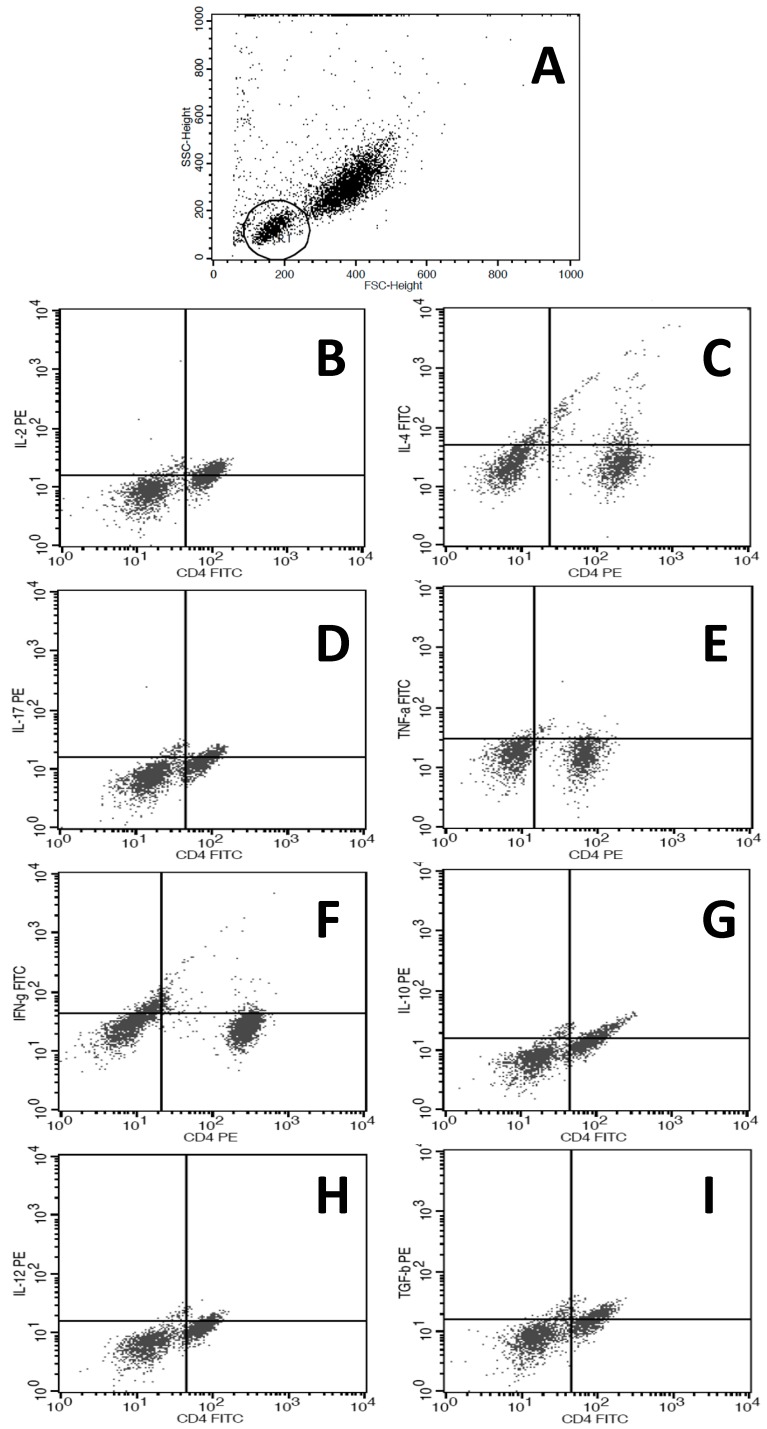

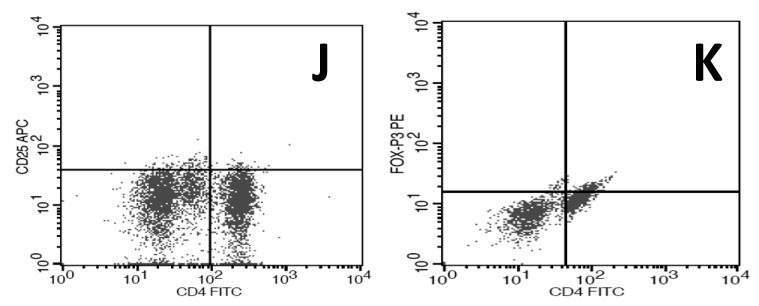
Representative the strategy for FACS analysis and a representative FACS figure. (**A**) The gate was determined for localization of cells corresponding to lymphocytes by for relative size (Forward Scatter, FSC) and granularity/complexity (Side Scatter, SSC) in each experiment and for each patient; (**B**–**K**) representative graphic obtained from one patient according with antibodies used, illustrating the positive cells: (**B**) CD4^+^IL-2^+^; (**C**) CD4^+^IL-4^+^; (**D**) CD4^+^IL-17^+^; (**E**) CD4^+^TNF-α; (**F**) CD4^+^IFN-γ^+^; (**G**) CD4^+^IL-10^+^; (**H**) CD4^+^IL-12^+^; (**I**) CD4^+^TGF-β^+^; (**J**) CD4^+^CD25; (**K**) CD4^+^FOXP3^+^.

Finally, the cells were resuspended in PBS (500 μL) and analyzed with a BD FACSCalibur™ cytometer. For an accurate determination of the cells corresponding to lymphocytes and no other cell type, we determined the region to be analyzed by constructing gates according to controls for relative size (Forward Scatter, FSC) and granularity/complexity (Side Scatter, SSC) in each experiment and for each patient. In Figure 3 we demonstrated the strategy for FACS analysis and a representative FACS figure for each immunomarker studied.

### 4.5. Statistical Analysis

An electronic database was developed for the statistical analysis. Variables were analyzed with the GraphPad Prism 4.0 program. Values were submitted to Mann-Withney or Kruskall Wallis test. Differences with *p* ≤ 0.05 were considered to be statistically significant.
